# The State Endowment of Motherhood

**Published:** 1916-05-06

**Authors:** 


### The State Endowment of Motherhood.

The Chairman of the Health Committee of the Bradford Corporation, Mr. E. J. Smith, has come out as a bold advocate of the State Endowment of Motherhood. Like others, he is anxious to see race suicide arrested, the calamitous decline in the birth-rate prevented, and the recognition of its obligations by the State to those who provide it with children is his remedy. Subject to certain conditions he suggests the endowment of motherhood with a national grant of five shillings a week until the child is able to earn its own living. The conditions Mr. Smith would wish to impose are these: (1) That the house is consistent with the physical and moral needs of the family in size and convenience; (2) that the home is kept reasonably clean; and (3) that a medical certificate should establish that the children are being properly fed, clothed, and cared for. "Along with this grant," he adds, "should go free ante-natal supervision, attendance on confinement, and after-care for both mother and child, subject to compliance with medical instructions and advice." In this way, it is urged, the birth-rate would begin to rise, infant mortality to fall, and the "damage rate" be abolished, while mothers would remain strong, vigorous, and healthy, and pass through the periods of expectancy and labour with infinitely less inconvenience and pain. In order to pay for the endowment of motherhood, Mr. Smith would impose a graduated tax, stopping at a figure at which hardship would be inflicted, upon single men and women and childless married couples. He would take the remainder of the necessary money out of the income-tax. The above rough outline of Mr. Smith's scheme brings out his main points, some of which are attractive, but it is hardly likely to receive sufficient sympathetic consideration at a period when war taxation will be making increasingly heavy demands upon the public purse.

